# Environmental drivers of reef manta ray (*Mobula alfredi*) visitation patterns to key aggregation habitats in the Maldives

**DOI:** 10.1371/journal.pone.0252470

**Published:** 2021-06-23

**Authors:** Joanna L. Harris, Guy M. W. Stevens

**Affiliations:** 1 The Manta Trust, Corscombe, Dorset, United Kingdom; 2 University of Plymouth, Plymouth, United Kingdom; Institut de Recherche pour le Developpement, FRANCE

## Abstract

A detailed understanding of the dynamics of small-scale (10s km) habitat use by the reef manta ray (*Mobula alfredi*) in the Maldives Archipelago is required to develop an effective national conservation management plan for this wide-ranging species. Here, a combination of photo-ID sightings data and acoustic telemetry were used to investigate both long-term *M*. *alfredi* visitation trends and small-scale movement patterns to key habitats on the eastern side of Baa Atoll (Hanifaru Bay feeding area, Dhigu Thila multifunctional site, and Nelivaru Thila cleaning station). All tagged and most of the sighted *M*. *alfredi* exhibited high affinity to the eastern side of Baa Atoll, where 99% of detections occurred, and 69% of individuals were re-sighted in multiple years. Sightings data suggests that visitation patterns may be associated with differences in habitat use by sex and maturity status. Boosted regression trees indicated that tag detection probability at Hanifaru Bay increased with increased westerly wind speed (>5ms^-1^) during the day, close to a new and full moon just after high tide, and when the tidal range was low. Interaction effects between predictors suggest that wind-driven oceanographic processes, such as Langmuir Circulation, maybe working to increase zooplankton concentration at this location. Tag detection probability increased at Dhigu Thila under similar conditions. At Nelivaru Thila, it increased at lower wind speeds (<5ms^-1^), close to a full moon, three hours after high tide. These results suggest that *M*. *alfredi* may utilise cleaning stations during the day when environmental conditions are not suitable for feeding. There was a high level of connectivity between these three locations, which suggests they form part of a network of key habitats that provide essential services to *M*. *alfredi* locally. Future conservation efforts should focus on identifying all areas of key habitat use for this species within the Maldives; applying strict protective measures to these sites and any connecting migration corridors which link them.

## Introduction

Reef manta rays (*Mobula alfredi*) are widely distributed throughout the tropical and sub-tropical waters of the Indo-West Pacific Oceans in relatively small, seemingly fragmented regional populations [1–3], which appear to be dependent on coral reef ecosystems [4]. Satellite tagging, acoustic telemetry and photographic identification studies have shown the species often exhibit high levels of residency and site fidelity whereby movement patterns are limited and individuals frequently return to the same location [4–13]. Site fidelity is associated with resource requirements [8], in particular at feeding areas, where high densities of zooplankton prey allow for energetically efficient foraging [14, 15], and at cleaning stations, where cleaner fishes remove parasites, and *M*. *alfredi* engage in social and reproductive interactions, and potentially to thermoregulate [15, 16]. At both feeding areas and cleaning stations, it is common for *M*. *alfredi* to aggregate in large numbers (>20) [7, 15, 17, 18]. At some locations, aggregation sites are utilised by *M*. *alfredi* year-round with seasonal peaks in the number of individuals present [12, 19–21]. At others, there are strong seasonal patterns in habitat use [5, 7, 13, 22].

Like other mobulid species, targeted and bycatch fisheries are the most significant anthropogenic threats to *M*. *alfredi*, particularly in the Indian Ocean [23], and extensive declines appear to be co-occurring with ongoing fishing pressure [19, 23, 24]. As a slow-growing species which is late to mature, producing only a few offspring during their lifetime, population recovery from exploitation is slow [15, 25]. Furthermore, other anthropogenic threats, such as habitat degradation, the climate crisis, and unregulated tourism pressure, are all likely to hinder survivorship [25–29]. Aggregation behaviour can be spatially and temporally predictable, potentially increasing their vulnerability to exploitation [7].The *M*. *alfredi* population of the Maldives Archipelago is the largest documented to date; engaging in a biannual migration which is influenced by the South Asian Monsoon [7, 30]. The South Asian Monsoon has two components (seasons), the Northeast (NE) Monsoon (*Iruvai*), which runs from December to March, and the Southwest (SW) Monsoon (*Hulhangu*), which runs from April until November [7]. In the NE Monsoon, *M*. *alfredi* frequent key habitats located around the shallow reef on the west side of the atolls, while during the SW Monsoon, they utilise habitats on the east [7]. The species are protected from targeted fisheries in the country following the Environmental Protection Agency’s 2014 law whereby it was made illegal to capture, keep, or harm any type of ray, as laid out in Batoidea Maldives Protection Gazette No. (IUL) 438-ECAS/438/2014/81 [31]. However, they are still at risk from other human activities. For example, fishing gear entanglement and vessel strikes [32] and disturbance from tourism activities which can disrupt natural behaviour [27]. As a highly mobile species, implementing protection throughout their range is challenging; instead, conservation measures need to be focused on important habitats where protection will be most beneficial [20, 23]. *Mobula alfredi* are particularly vulnerable to disturbance at aggregation sites where the species come to feed or engage in cleaning and social and reproductive activities [15, 16]. At feeding areas, *M*. *alfredi* can abandon feeding when their path is obstructed by visitors [27] and lethal and sublethal injuries can be caused by fishing gear entanglement, boat strikes and propeller injuries [32]. At cleaning stations, anthropogenic activities, such as the destruction of coral reefs for boat access or contact damage by divers and snorkellers, can lead to habitat degradation [32], potentially influencing *M*. *alfredi* visitation patterns [33] and compromising individual fitness [34].

Some of the fundamental components required to identify areas of conservation concern which should be prioritised for protection in the Maldives have recently been studied and addressed [7]. These include the determination of important *M*. *alfredi* habitats, knowledge of the species broad-scale spatiotemporal distribution, and an understanding of the drivers of these patterns [7]. The aforementioned study indicated that *M*. *alfredi* distribution patterns were influenced by the onset and retreat of the SW Monsoon winds and increased chlorophyll-a (productivity) associated with nutrient upwelling on the down-current side of the atolls. During the SW Monsoon, the wind drives ocean currents eastwards, enhancing productivity off the eastern edge of the archipelago’s atolls. The retreat of the SW Monsoon for part of the year then gives way to the NE Monsoon winds, which enhance productivity off the western edges of the atolls during the NE Monsoon. However, to be able to effectively manage human disturbance at specific locations, a more detailed understanding of the dynamics of small-scale (10s km) habitat use is required. Therefore, the current study focuses primarily on the east side of Baa Atoll, where various key habitats, utilised predominantly during the SW monsoon, have been identified and highlighted as in need of greater protection [7]. Using photo identification, acoustic telemetry and modelling techniques, this study aims to: 1) investigate small-scale movement patterns of *M*. *alfredi*, 2) examine visitation patterns to key habitats (feeding areas and cleaning stations), and 3) identify and compare environmental and temporal influencers of *M*. *alfredi* visitation patterns to and between key aggregation sites during the SW monsoon.

## Materials and methods

### Study location

**Fig 1:**
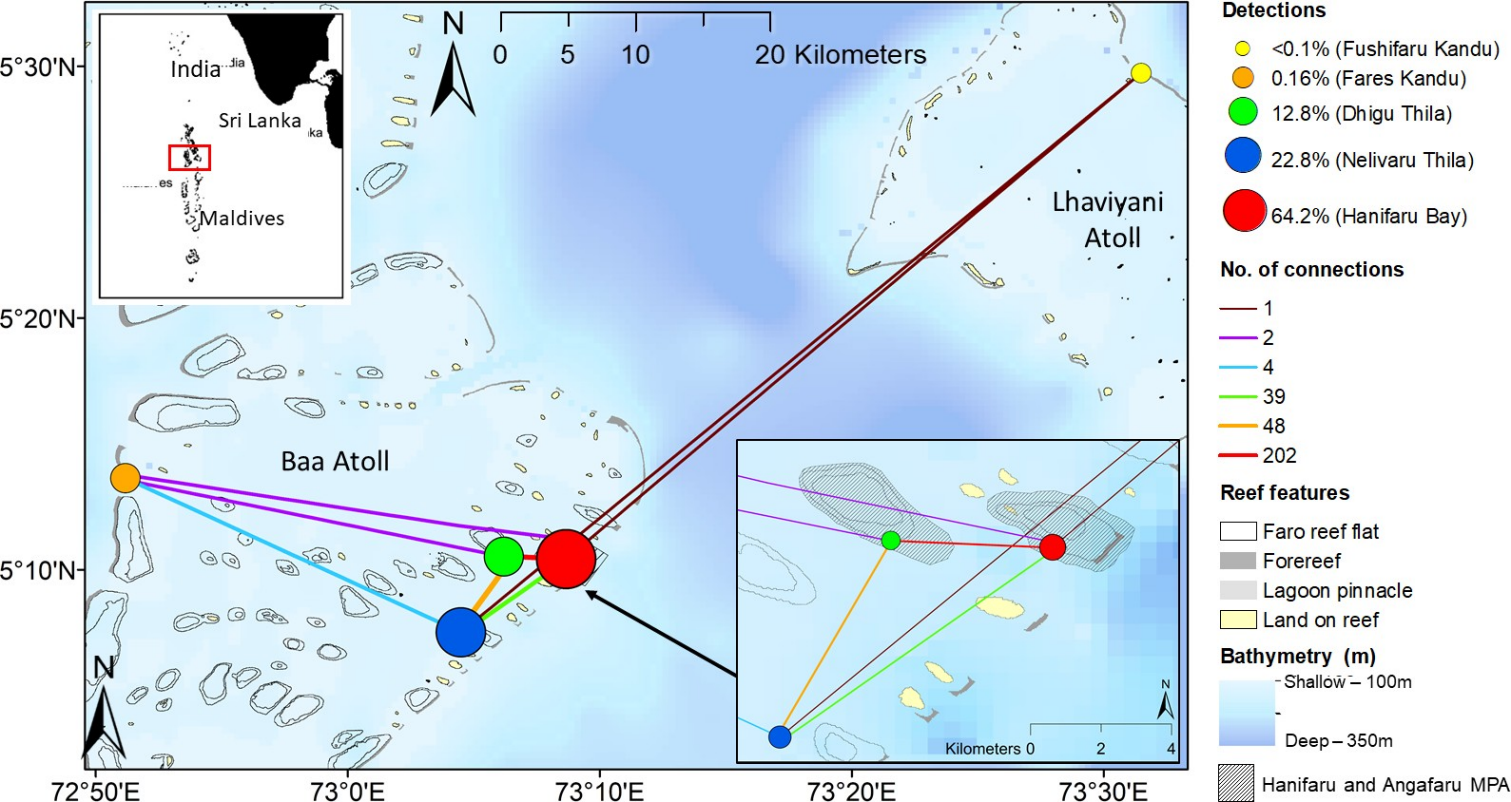
*Mobula alfredi* tag detections and acoustic receiver network map. Percentage of detections at each site corresponds to the colour and size of the node at each location. The network map showing how many times individuals were subsequently detected by each pair of acoustic receivers which corresponds to the colour and thickness of the line connecting the nodes. Map created in ArcGIS 10.7 (www.esri.com/) using bathymetry from GEBCO Compilation Group (2020) GEBCO 2020 Grid (doi:10.5285/a29c5465-b138-234d-e053-6c86abc040b9) and reef features from Millennium Coral Reef Mapping Project (MCRMP) (https://data.unep-wcmc.org/datasets/1); MCRMP validated maps provided by the Institute for Marine Remote Sensing, University of South Florida (IMaRS/USF) and Institut de Recherche pour le Développement (IRD, Centre de Nouméa), with support from NASA. MCRMP unvalidated maps provided by IMaRS, USF with support from NASA and further interpreted by UNEP World Conservation Monitoring Centre (www.unep-wcmc.org). IRD, do not endorse these products. Inset top left: the Maldives Archipelago with the study area highlighted in the red box. Inset bottom right: close-up of the key habitats (Hanifaru Bay, Nelivaru Thila and Dhigu Thila) and the Hanifaru and Angafaru marine protected areas (MPAs).

Straddling the equator, the Maldives Archipelago is comprised of 26 geographical coral atolls situated mostly in the northern Indian Ocean. The current study focuses on five sites across Baa and Lhaviyani Atolls (Fig 1).

#### Hanifaru Bay, Baa Atoll

Hanifaru Bay is a small (700 x 200 m) and shallow (22 m max depth) reef inlet with a sandy seabed situated on the east side of Baa Atoll. It is a cul-de-sac ‘bay’ surrounded by shallow (<1 m) reef along all but a small 75 m section of the Bay’s circumference, situated at the western end. Hanifaru is a key aggregation site for *M*. *alfredi*, which frequent this site primarily to feed during the SW Monsoon [7, 15].

#### Dhigu Thila, Baa Atoll

Dhigu Thila, situated on the east side of Baa Atoll, is a long narrow (550 x 15 m) reef that rises steeply from the Baa Atoll’s sandy lagoonal seabed (45 m) to within 7 m of the surface at its shallowest easternmost point, averaging 12 m along the reef crest. Dhigu Thila is a key aggregation site for *M*. *alfredi*, which frequent this site primarily to clean and feed during the SW Monsoon [7, 15].

#### Nelivaru Thila, Baa Atoll

Nelivaru Thila, situated on the east side of Baa Atoll. is a small triangular-shaped reef (250 x 100 m) that rises steeply from Baa Atoll’s sandy lagoonal seabed (45 m) to within 2 m of the surface at its shallowest point, averaging 5 m along the reef crest. Nelivaru Thila is a key aggregation site for *M*. *alfredi*, which frequent this site primarily to clean during the Southwest Monsoon [7, 15].

#### Fares Kandu, Baa Atoll

Fares Kandu is 1.5-kilometre-wide channel situated on the west coast of Baa Atoll. Inside the channel, the depth of the seabed reaches 40 m. During the NE Monsoon, *M*. *alfredi* feed and visit cleaning stations at this site.

#### Fushifaru Kandu, Lhaviyani Atoll

Fushifaru Kandu is 600-metre-wide channel situated on the northeast coast of Lhaviyani Atoll. Inside the channel, the depth of the seabed reaches 45 m. During the SW Monsoon, especially during October, *M*. *alfredi* feed and visit cleaning stations inside the channel.

### Tag deployment

Acoustic tags (Vemco; n = 13) were externally deployed on *M*. *alfredi* in Baa Atoll across two tagging periods; August 2007 (Period One; n = 4) and September 2009 (Period Two; n = 9) (Table 1). All tags deployed in Period One were V16-5H tags, and during Period Two, 13 V16-5H and 9 V16TP-5H (temperature and depth sensor tags) were deployed. Tag anchors were made of stainless steel, and all were of a similar design to the large titanium anchor (Wildlife Computers). Tags were deployed *in situ* using a modified Hawaiian sling and positioned towards the posterior dorsal surface of each manta ray. Positioning ensured the anchor did not pierce the body cavity and the tag was not trailing over the back edge of the pectoral fin. All tagged animals were photographed and identified prior to being tagged, and their size and sex recorded with the tag ID number. All 13 tagged *M*. *alfredi* were sighted or detected on multiple occasions after tag deployment in subsequent days, months, and years. All were behaving naturally when observed, with no adverse signs of injury or behavioural change resulting from the tagging. Tagging activities were conducted under ethics approval and permit from the Maldives’ government (permit numbers: FA-D2/33/2007/07 and FA-D2/33/2009/03).

**Table 1:** Acoustic tag deployment summary.

Manta ID	Tag ID	Sex	Maturity	Deployment location	Tag Deploy Date	Date of last detection	No. detections	No. of sites	No. tracking days	No. detection days	Residency Index (I_R_)	I_R_—Baa Atoll
MV-MA-0057	32718	F	Juvenile	Hanifaru Bay	29/09/2009	26/11/2009	1049	2	59	27	45.80%	45.80%
MV-MA-0283	32714	F	Adult	Hanifaru Bay	22/11/2009	05/06/2010	923	3	196	24	12.20%	12.20%
MV-MA-0414	32711	F	Adult	Hanifaru Bay	29/09/2009	01/10/2009	52	1	3	2	N/A	N/A
MV-MA-0447	2390	F	Adult	Hanifaru Bay	13/09/2009	23/09/2010	5142	4	376	94	25.00%	24.70%
MV-MA-0783	2393	F	Adult	Dhigu Thila	29/08/2007	30/09/2007	138	2	33	3	9.10%	9.10%
MV-MA-0793	2392	F	Adult	Dhigu Thila	31/08/2007	28/10/2007	1647	3	59	37	62.70%	62.70%
MV-MA-0842	2388	M	Adult	Hanifaru Bay	29/08/2007	30/08/2007	4	3	2	2	N/A	N/A
MV-MA-0850	2394	F	Adult	Dhigu Thila	31/08/2007	19/11/2008	4937	3	447	109	24.40%	24.40%
MV-MA-0901	32719	F	Juvenile	Hanifaru Bay	19/09/2009	20/06/2010	721	4	275	29	10.50%	9.50%
MV-MA-1217	2389	F	Adult	Hanifaru Bay	12/09/2009	13/10/2009	2509	2	32	21	65.60%	65.60%
MV-MA-1260	32712	F	Adult	Hanifaru Bay	29/09/2009	10/09/2010	2255	4	347	64	18.40%	17.90%
MV-MA-1365	32713	F	Adult	Hanifaru Bay	13/09/2009	13/07/2010	3914	4	304	78	25.70%	23.70%
MV-MA-1739	2395	F	Adult	Hanifaru Bay	12/09/2009	09/08/2010	3327	3	332	76	22.90%	22.90%

Acoustic tags deployed on to reef mantas at Hanifaru Bay and Dhigu Thila (c.a. 5.17, 73.14) in the Maldives Archipelago during 2007 and 2009. No. detections: total number of detections between the date of the first and last detection; No. tracking days: total number of days between first and last detection; No. detection days: total number of days the individual was detected; Residency Index (I_R_): 0% (no residency) and 100% (absolute residency).

### Acoustic receiver array

An array of five omnidirectional acoustic receivers (VR2W-69 kHz, Vemco Inc., Nova Scotia, Canada) were deployed around Baa and Lhaviyani Atolls in the Maldives Archipelago (Table 2). Three were positioned in August 2007 at key habitats in eastern Baa Atoll: Hanifaru Bay, Nelivaru Thila and Dhigu Thila [7]. Recording ceased in November 2008 when the acoustic receivers were retrieved. They were then redeployed in September 2009, when two further receivers were also added to extend the array to Fares Kandu in western Baa Atoll and at Fushifaru Kandu in eastern Lhaviyani Atoll. All receivers were positioned within the atoll’s lagoons, suspended approximately 2 m above the seabed at depths between 21 m and 41 m. Deployed tags were detected within approximately 150 m of the receivers as determined by preliminary range testing (mean = 165 ± 33 m) [35]. Data from receivers were downloaded every 6–12 months. Once a year, the battery of each receiver was replaced, and the receiver inspected for damage or clock drift.

**Table 2:** Summary of acoustic receiver deployment.

Site	Deployed	Depth	Retrieved	No. days recording	No. *M*. *alfredi* detected	Detections (%)	Mean resident event (mins±SD)	Max resident event (mins)
Dhigu Thila	23/08/2007	41	11/08/2010	791	12	12.84%	28 ± 44	275
Hanifaru Bay	23/08/2007	22	26/08/2010	808	12	64.21%	119 ± 126	822
Nelivaru Thila	27/08/2007	31	01/11/2010	866	10	22.78%	71 ± 81	509
Fares Kandu	26/09/2009	21	12/12/2010	443	3	0.16%	10 ± 14	43
Fushifaru Kandu	13/12/2009	26	26/09/2010	288	1	0.01%	-	3

Acoustic receiver deployment details with detections and resident events. Acoustic receivers were not recording at Hanifaru Bay, Dhigu Thila and Nelivaru Thila between November 2008 and September 2009 (approximately 300 days).

### Range test and tag detectability

Range tests were performed on the acoustic receivers at Hanifaru Bay and Dhigu Thila by deploying an acoustic transmitter tag attached to an anchor and rope, suspended one meter below the surface. At both sites, the tag was deployed for 45 minutes at six different locations up to 200 m from the acoustic receiver. Detection records were then used to determine the maximum distance a reliable detection occurred. At both sites, the tag was successfully detected at all six locations.

Detections from the transmitter deployed on manta MV-MA-1365 became continuous on 13^th^ July 2010 at Hanifaru Bay as the tag had been shed within the receiver’s range. The authors confirmed the loss of the tag when the individual was sighted near the receiver on the same day with a fresh, post-tag-shed wound. Detection data from the shed tag was used as a ‘sentinel tag’ [6] to investigate the influence environmental variables have on tag detectability [6]. Couturier *et al*. [6] suggested that even without knowing the exact position of the tag in relation to the acoustic receiver, as is the case here, testing the incorporation of the detection data from the sentinel tag into the models can assist in assessing the reliability of the *M*. *alfredi* presence data.

### Acoustic detection analysis

Detection data were imported to VUE software (version 2.6.2) and receiver clock -drift was corrected, assuming linear drift [20]. False detections of tags were removed from analysis by first filtering for active tags and then applying the False Detection Analyser using the default short to long periods between detections, <30 min and >12 h, respectively [36]. Any tag which was continuously detected by a single receiver for >24hrs were considered to have detached within the receivers range and were also removed [20]. The VTrack R package [37] in R 3.5.2 [38] was then used to summarise detection data.

Visitation frequency at all five locations was assessed by constructing a network map using the ’igraph’ R package [39] in R 3.5.2 [38]. Briefly, each acoustic receiver represents a ’node’. The size of the node shows the percentage of detections which occurred at that location. Each node is connected by edges which represent the frequency of which tagged individuals travelled between pairs of receivers [20]. The resulting network map was projected in ArcGIS 10.7.

To compare overall residency of individuals within the whole acoustic array to that of key habitats only (Hanifaru Bay, Nelivaru Thila and Dhigu Thila), residency indices (I_R_) were calculated using the following form adapted from Afonso *et al*. [40]. The results range from 0% (no residency) and 100% (absolute residency) [40]. To reduce bias that might arise when including individuals with a low number of tracking days, only those with >30 tracking days were included in this analysis.


$$I_{R}=\frac{Number\;of\;days\;detected}{Number\;of\;days\;between\;first\;and\;last\;detection+1}\;x\;100$$

To determine the intensity at which each location was utilised, the amount of time each individual spent within the detection range of each acoustic receiver was calculated using the VTrack R package [37]. All detections of individual tags are classed as either ’non-resident’ and ’resident events’. Here, a non-resident event occurs when a tag was only detected by an acoustic receiver on one occasion within 60 minutes. A resident event started when there were two or more successive detection at the same receiver [41] within 60 minutes. The resident event then terminated at the time of the last detection when there were no further detection within 60 minutes, or when the transmitter was detected at least twice at another receiver [37, 41].

### Environmental modelling

Boosted regression trees (BRT) were used to investigate the relationship between environmental variables and the visitation patterns of tagged *M*. *alfredi* at the identified feeding area (Hanifaru Bay), cleaning station (Nelivaru Thila), and multifunctional site (feeding and cleaning, Dhigu Thila). The technique uses a model averaging (ensemble) method that allows for both explanation and prediction [42]. Many, relatively simple, models are fitted and then combined for prediction using two algorithms: regression trees and boosting [42]. The regression tree is constructed from predictor variables through a series of binary splits [43]. Splits occur based on the homogeneity of the predictor variables relationship to the response variable [44]. Multiple possible splits are tested by the algorithm, and partitioning occurs when the greatest improvement of homogeneity is found [44]. Subsequent trees are then fitted sequentially to the residuals from the previous ones [45]. The technique is advantageous as it can fit complex, non-linear relationships, and models interactions between response variables [42]. Model building requires specification of various parameters: tree complexity (*tc*), which specifies the number of interactions that should be modelled [42], learning rate (*lr*), which regulates the contribution of each tree to the growing model [42], bag fraction (*bf*), which controls stochasticity by randomly selecting (without replacement) a specified subset of the data at each iteration [42], and step size (*ss*), which controls the number of trees which should be added at each iteration.

To identify the influences of small-scale (10s km) habitat use which occur at three key habitats (Hanifaru Bay, Nelivaru Thila and Dhigu Thila) during the SW Monsoon (when they are predominantly utilised by *M*. *alfredi* [7]), all detection data from the SW Monsoon months (August-November 2007, April-November 2008, September 2009-November 2009 and April 2010-September 2010) were pooled by location and a binomial response variable of present (1) and absent (0) was established for each hour; from five hours before the first detection until five hours after the last detection during the period while acoustic receivers were recording (Table 2). The final time-series for each location included the following number of presence and absence observations: Hanifaru Bay = 13335, Dhigu Thila = 12971, and Nelivaru Thila = 13470.

Six predictor variables which have been shown to influence *M*. *alfredi* occurrence were selected for inclusion: hour of the day, moon illumination, time relative to high tide, hourly tidal range, hourly wind speed, and hourly wind direction [5, 6, 20]. Hour of the day corresponds to the hour in which the detection occurred, as recorded by the acoustic receiver. Moon illumination data was provided by the United States Naval Observatory (http://aa.usno.navy.mil/data/docs/MoonFraction.php). Tides were modelled using the Oregon State University Tidal Model Driver (TMD) [20, 46] based on semidiurnal (M2 and S2) and diurnal (K1 and O1) tidal constituents [47]. Time relative to high tide was then calculated as negative hours before (flood) and positive hours after (ebb) with high tide as zero [20]. Hourly tidal range is the hourly change in tide height (m) relative to mean sea level as modelled by TMD. Hourly wind speed and direction data (10m above ground) were obtained from Meteoblue AG, Basel, Switzerland (www.meteoblue.com).

Collinearity between predictor variables was assessed using pairwise correlation coefficients and variance inflation factor (VIF) estimates [45]. None of the predictor variables were highly correlated, i.e. no coefficients were >0.6, and all VIF estimates were <3.5 [45] (S1 and S2 Tables and S1–S4 Figs).

The BRT models were fitted with Bernoulli distribution using the gbm.step() function of the dismo R package [48]. Trees were built with recommended parameters to ensure model outputs were comparable among locations [45]: *tc* of 5, *lr* of 0.05, *bf* of 0.5 and *ss* of 50 [49]. All combinations of parameters *tc (1*, *2*, *3*, *4*, *5)*, *lr* (0.01, 0.005, 0.001 and 0.0001), *bf* (0.5, 0.7, 0.9) and *ss* (25, 50) [45, 50] were also tested, but provided minimal improvement in model performance.

Model performance was assessed via ten-fold CV [42], which tests the model (primary sample) against a withheld subset of the data (hold-out sample), which is not used for model fitting [45]. The model’s ability to fit the data was then measured using area under the receiver operating characteristic curve (AUC) test statistic [51, 52]. The AUC test statistic is a threshold-independent metric which reflects the model’s ability to accurately classify observations [53]. Each model output contains two mean AUC values, one for its performance classifying the primary sample (training AUC, TAUC) and another for the hold-out sample (cross-validation AUC, CVAUC) [49, 52]. The AUC classification ranges from 0–1, whereby values >0.7 are acceptable, 0.8–0.9 are excellent, and those >0.9 are outstanding [54]. The level of overfitting of the primary sample is indicated by the difference between the TAUC and the CVAUC (ΔAUC) [52]. Therefore, better model performance is categorised by higher AUC values for both TAUC and CVAUC but a lower ΔAUC [52]. To quantify how well the models fitted the data, the pseudo determination coefficient percentage of deviance explained (*D*^2^) was calculated using the following form [55].


$$D^{2}=1-(residual\;deviance/total\;deviance)$$

Model results reflect the relative influence of predictor variables, which is measured by averaging the number of times a variable is chosen for splitting and the squared improvement resulting from these splits [42]. The result is then scaled to 100 across all the variables [42]. The predictor variables with the highest numbers indicate a more substantial influence on the response variable [42].

Partial dependency plots were generated using an author-modified version of the gbm.plot() function. The plot represents the effect of the explanatory variable after accounting for the mean effects of all other explanatory variables [43]. Confidence intervals (95%) for the partial dependency plots were obtained from 1000 bootstrap replicates [45].

The relative Interaction strengths between predictor variables were quantified by measuring residual variation between pairwise model predictions with and without interactions [42, 45, 56], while holding all other variables to their respective mean [42]. One hundred bootstrap resampling was used to test the significance of the strongest interactions [45, 56]. The technique was applied via the ggInteract_boot() function of the ggBRT R package [57], which randomly sampled the occurrence of *M*. *alfredi* at each location before re-fitting the BRT models [45]. The size of the interaction is then recorded to generate a distribution under the null hypothesis of no interaction among predictors [45].

To investigate potential changes in tag detectability which might be caused by the environmental variables considered here, the same method was applied to two further models constructed for Hanifaru Bay using a subset of data from the time period that the sentinel tag was transmitting until the acoustic receiver was retrieved on 26^th^ August 2010. There were 32659 detections during this 43-day period. Both models contained 1045 hourly presence and absence observation and the six explanatory variables. One model also included the hourly detection count of the sentinel tag as an additional explanatory variable [6]. These models were compared based on their performance (whether the addition of hourly detection count of the sentinel tag improved model performance) and the variation in the influence of the explanatory variables and interaction effects with and without the hourly detection count of the sentinel tag.

### Photographic identification

The Manta Trust’s Maldives Manta Conservation Program photo identification (photo-ID) database (www.mantatrust.org/idthemanta) was used to broadly assess long-term *M*. *alfredi* visitation patterns to key habitats. Data were recorded during systematic surveys conducted by trained Manta Trust researchers and volunteers between 2005–2019 (see Harris *et al*. (2020) for details of the full photo-ID methodology). Statistical analysis was not performed on these data because of survey bias (e.g., surveys are only conducted during the day and survey effort is not consistent across all sites). Instead, sightings (a confirmed photo-ID of an individual *M*. *alfredi* on a given day at a defined location [7]) were summarised to provide insight into *M*. *alfredi* habitat use by demographics and annual visitation patterns.

## Results

### Detection and residency summary

For all 13 of the acoustic tags deployed (Table 1), there were a total of 26618 detections recorded. Of these, 64.2% (17091) occurred at Hanifaru Bay, 22.8% (6064) at Nelivaru Thila, and 12.8% (3418) at Dhigu Thila. Less than 1% of detections occurred at Fares Kandu (42) and Fushifaru Kandu (3). On average, tags were retained for 190 ± 163 days (range 2–447) with a mean of 44 ± 37 detection days (range 2–109). Residency indices show that individuals were detected within the array for a mean of 29% of the days they were tracked (I_R_ = 29.3 ± 19.9%), with a minimum and maximum I_R_of 9.1% and 65.6%, respectively (Table 1). Residency indices calculated for the key habitats only (Hanifaru Bay, Nelivaru Thila and Dhigu Thila) show that individuals were detected in the array for a mean of 29% of the days they were tracked (I_R_ = 28.9 ± 20.1%).

Twelve of the tagged *M*. *alfredi* visited Hanifaru Bay and Dhigu Thila, and ten visited Nelivaru Thila, while only one and three individuals visited Fushifaru Kandu and Fares Kandu, respectively. Hanifaru Bay and Dhigu Thila were the pair of acoustic receivers that were most frequently connected by subsequent detections, followed by Hanifaru Bay and Nelivaru Thila (Fig 1).

**Fig 2:**
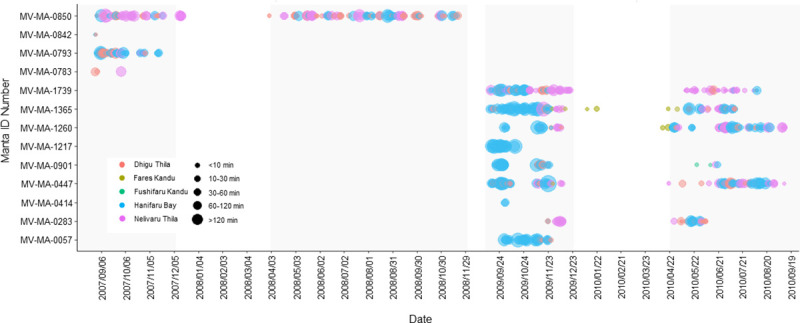
Resident events recorded at each location. Resident events at each site showing location (by colour) and time at location (by size). Grey shading highlights the SW Monsoon. Note the gap in the timeline between 29/11/2008 and 08/09/2009 when the acoustic receivers were not recording.

Overall, there were 857 resident events recorded for 12 *M*. *alfredi* (Fig 2). At Hanifaru Bay, there was a total of 384 resident events altogether totalling 47790 mins (770.5 hrs). Individual mean resident event time at this location was 119 ± 126 mins. The longest resident event at this location was 822 min (13.7 hrs) (manta-ID MV-MA-0447). At Dhigu Thila, 269 residency events occurred totalling 8695 mins (144 hrs), with a mean resident event time of 28 ± 44 mins. The longest resident event at this location was 275 mins (4.6 hrs) (manta-ID MV-MA-0447). Nelivaru Thila had a total of 195 resident events totalling 15335 mins (255.5 hrs), with a mean resident event time was 71 ± 81 mins, and the longest event was 509 mins (8.5 hrs) (manta-ID MV-MA-1365). Fares Kandu had eight resident events totalling 87 mins with a mean resident event time of 10±14 mins and a maximum time of 43 mins (manta-ID MV-MA-1260). At Fushifaru Kandu, there was only one resident event, which lasted for three minutes (manta-ID MV-MA-0901).

Of the 857 resident events recorded, 813 (95%) occurred during the SW Monsoon (April–November). Of the 44 resident events which occurred during the NE Monsoon (December–March), two were at Dhigu Thila, two at Fares Kandu, and 40 occurred at Nelivaru Thila. Apart from one event at Fares Kandu, which occurred in January, all the events during the NE Monsoon occurred during March and December, which are monsoon transition periods.

**Fig 3:**
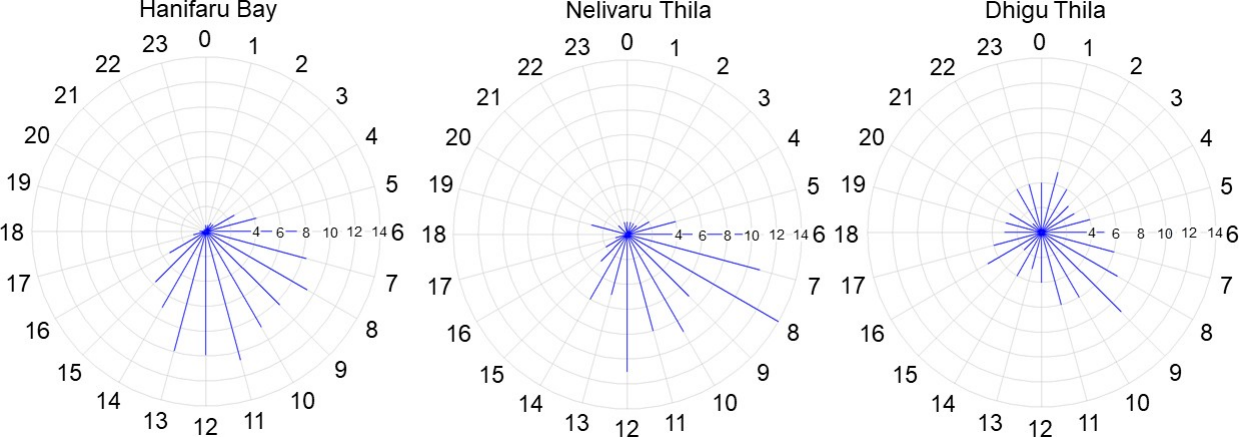
Distribution of resident events by hour of the day. Percentage distribution of resident events (start time) by hour of the day for Hanifaru Bay, Nelivaru Thila, and Dhigu Thila. Data for Fushifaru Kandu and Fares Kandu not shown as only nine resident events occurred between these locations.

The overall distribution of resident events by hour of the day for the entire acoustic array shows that 81% occurred during the day (sunrise and sunset to the nearest hour, 06:00–18:00) (Fig 3). There was only a single resident event at Fushifaru Kandu which occurred at night, at all other locations resident events occurred more frequently during the day: Hanifaru Bay = 90.1%, Nelivaru Thila = 88.2%, Fares Kandu = 75%, and Dhigu Thila = 62.8%. Of the night-time events which occurred at Hanifaru Bay (37 events), 70% occurred in the hours approaching sunrise (04:00–05:00) (Fig 3), and all lasted into daylight hours. At Nelivaru Thila, 23 night-time events occurred, of which 57% did not span daylight hours. Night-time events occurred most frequently at Dhigu Thila, where 100 (37.2%) resident events occurred between 19:00–05:00, none of which spanned daylight hours. At Fares Kandu, only two night-time events occurred, one between 01:26–01:44 and the other between 20:33–20:37.

More than 30 resident events were recorded for ten of the thirteen tagged *M*. *alfredi*. The percentage of resident events which occurred at each location varied considerably between individuals. For example, 90.3% of resident events for manta MV-MA-1271 occurred at Hanifaru Bay, while this manta was not detected at Nelivaru Thila. In contrast, 51% of resident events for MV-MA-1739 occurred at Nelivaru Thila, and only 30% occurred at Hanifaru Bay. Resident events for manta MV-MA-0793 predominantly occurred at Dhigu Thila (47.95%). Resident events for manta MV-MA-0850 also occurred most frequently at Dhigu Thila (44.1%), while only 23% occurred at Hanifaru Bay.

## Environmental influences: Boosted Regression Trees (BRT)

### Hanifaru Bay feeding area

Model performance evaluation of the Hanifaru Bay BRT shows the final model had outstanding predictive performance for the training (_T_AUC = 0.99) and cross-validated data (_CV_AUC = 0.90), with minimal evidence of overfitting (ΔAUC = 0.08). The estimated *D*^2^ suggests that 62.4% of the overall deviance was explained by the model.

**Fig 4:**
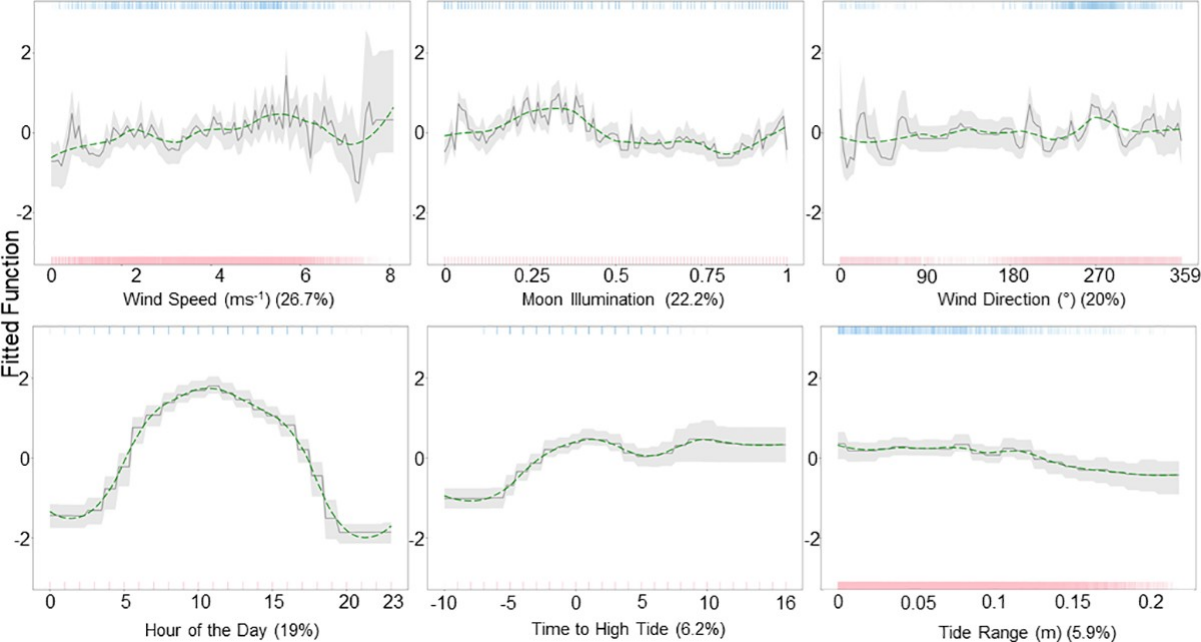
Partial dependency plots for Hanifaru Bay. The effect of each predictor variable on the occurrence of tagged *M*. *alfredi* at Hanifaru Bay, while keeping all other variables at their mean. The green dashed line shows locally weighted smoothing (LOESS). Rugs display the distribution of the data for presence (top, blue), and absence (red, bottom).

The relative influence of the predictor variables (Fig 4) indicates that wind speed (26.7%), moon illumination (22.9%), wind direction (20%), and hour of the day (19%) were the most accurate predictors of tag detections at Hanifaru Bay. The probability of tag detections increased with wind speed, peaking at moderate speeds (approximately 5ms^-1^). Detection probability then decreases before increasing at the highest speeds observed (8ms^-1^). Detection probability was highest with winds from 265° (westerly), after a new moon and close to a full moon, and during the day with a peak at 11 am. Although time to high tide (6.2%) and tide range (5.9%) had a relatively low contribution, they indicate a higher probability of detection just after high tide, and when the tide range is low, respectively.

**Fig 5:**
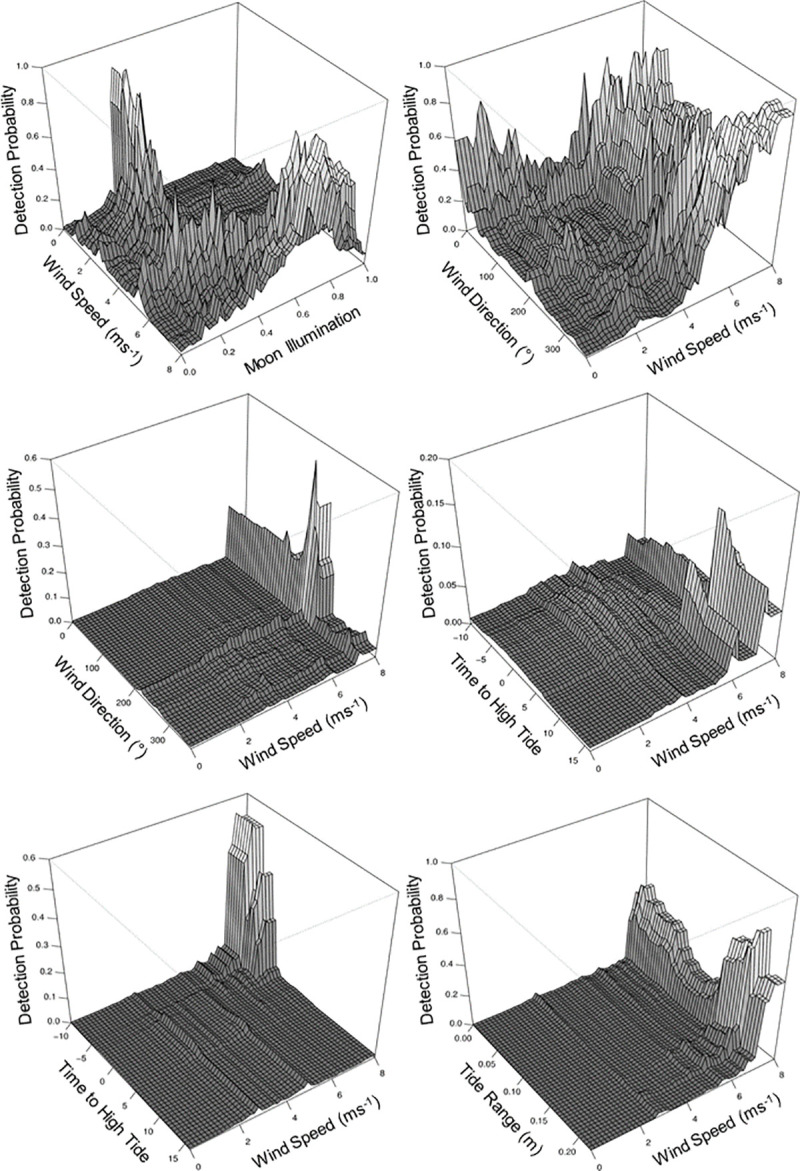
Interactions between predictor variables for all three locations. Pairwise interactions between predictor variables while keeping all other variables at their respective mean showing the probability of *M*. *alfredi* tag detections at Hanifaru Bay (top row), Dhigu Thila (middle row), and Nelivaru Thila (bottom row). All interactions were significant (*p*<0.01). Note z-axis scale varies depending on the influence of the interaction.

Significant interactions (*p*<0.01) occurred between wind speed and moon illumination, and wind speed and wind direction (S3 Table and Fig 5). They suggest that the probability of tag detections increased before a half-moon at low wind speed and before a full moon at high wind speeds. High wind speeds when the wind direction was approximately 100–120° and 285–350° also increase the probability of tag detections.

### Tag detectability

Performance of the model without the hourly detection count of the sentinel tag variable had outstanding (_T_AUC = 0.99) and excellent (_CV_AUC = 0.89) predictive performance for the training and cross-validated data, respectively, with minimal evidence of overfitting (ΔAUC = 0.1). The estimated *D*^2^ suggests that 69% of the overall deviance was explained by the model. Performance of the model with hourly detection count of the sentinel tag variable had marginally lower _CV_AUC (0.88) and *D*^2^ (66%). The relative influence of the six predictor variables was consistent in both models, which indicate that hour of the day, wind speed, moon illumination and wind direction were the most accurate predictors of tag detections. In the model that included the hourly detection count of the sentinel tag, this variable was the least accurate predictors of tag detections. The difference in percentage contribution of all other variable between models varied between 0.2–3.3% with the highest difference occurring between wind speed (S5 Fig).

There were two significant (*p*<0.01) interactions which were the same for both models, 1) hour of the day and wind speed where tag detection probability peaked at lower wind speeds during the day, with a smaller peak in probability at higher wind speeds (S6 Fig) and 2) time to high tide and moon illumination where the highest tag detection probability occurred close to a full moon just after high tide. The strength of these interactions was reduced in the model, which included hourly detection count of the sentinel tag most notably for the interaction, which included wind speed (S3 Table). No significant interaction occurred between hourly detection count of the sentinel tag and any other variable.

### Dhigu Thila multifunctional site

Model performance evaluation of the Dhigu Thila BRT shows the final model had outstanding and acceptable predictive performance for the training (_T_AUC = 0.95) and cross-validated data (_CV_AUC = 0.75), respectively, with some evidence of overfitting (ΔAUC = 0.2). The estimated *D*^2^ suggests that 38.5% of the overall deviance was explained by the model.

**Fig 6:**
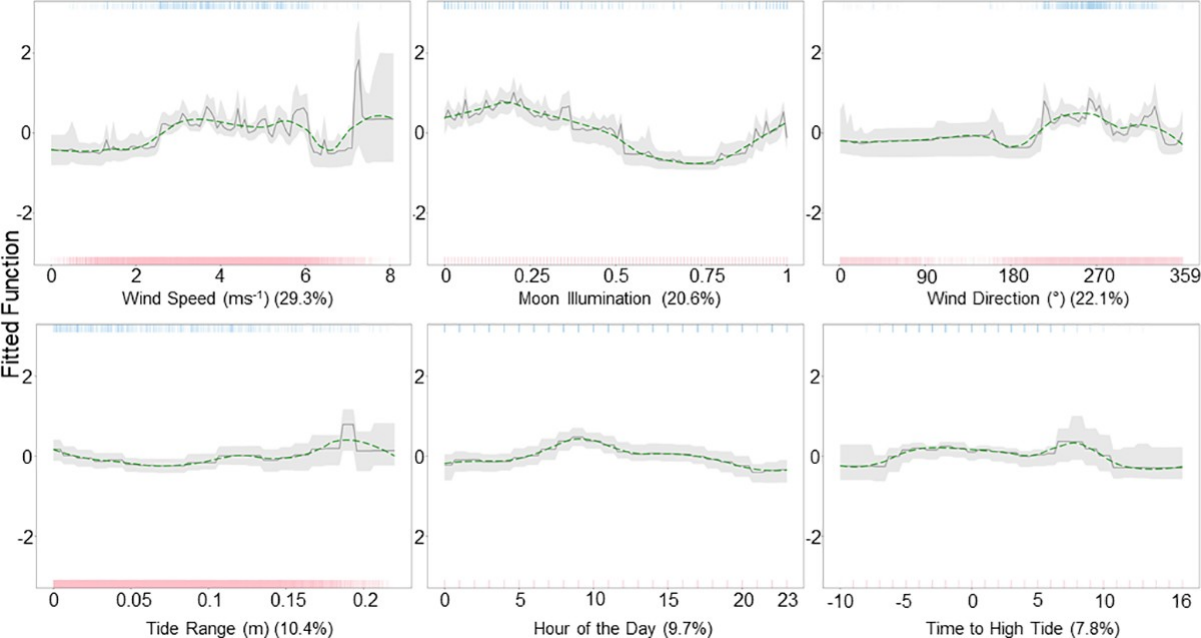
Partial dependency plots for Dhigu Thila. The effect of each predictor variable on the occurrence of tagged *M*. *alfredi* at Dhigu Thila while keeping all other variables at their mean. The green line shows locally weighted smoothing (LOESS). Rugs display the distribution of the data for presence (top, blue), and absence (red, bottom).

The relative influence of the predictor variables (Fig 6) indicates that wind speed (39.3%), moon illumination (20.6%), and wind direction (22.1%) are the most accurate predictors of tag detections at Dhigu Thila. The probability of tag detections increased with moderate wind speed (approximately 3 ms^-1^ and 6 ms^-1^), with a peak at higher wind speeds (approximately 7 ms^-1^). Detection probability was greatest after a new moon, and with winds from approximately 270° (W). Less influential variables included tide range (10.4%), hour of the day (9.7%), and time to high tide (7.8%), which suggest detection probability increases with tide range, during the day (with a peak between 9 am-10 am), and 6 hours after high tide, respectively.

Significant interactions occurred between wind speed and wind direction and wind speed and time to high tide (S3 Table and Fig 5). They suggest that the probability of tag detections increased at with wind speeds between 7–8 ms^-1^ when the wind direction was S–SSW (approximately 180–210°), and at wind speeds of 7–8 ms^-1^ approximately 5–6 hours after high tide.

### Nelivaru Thila cleaning station

Model performance evaluation of the Nelivaru Thila BRT shows the final model had outstanding and excellent predictive performance for the training (_T_AUC = 0.99) and cross-validated data (_CV_AUC = 0.88), respectively, with minimal evidence of overfitting (ΔAUC = 0.11). The estimated *D*^2^ suggests that 62.4% of the overall deviance is explained by the model.

**Fig 7:**
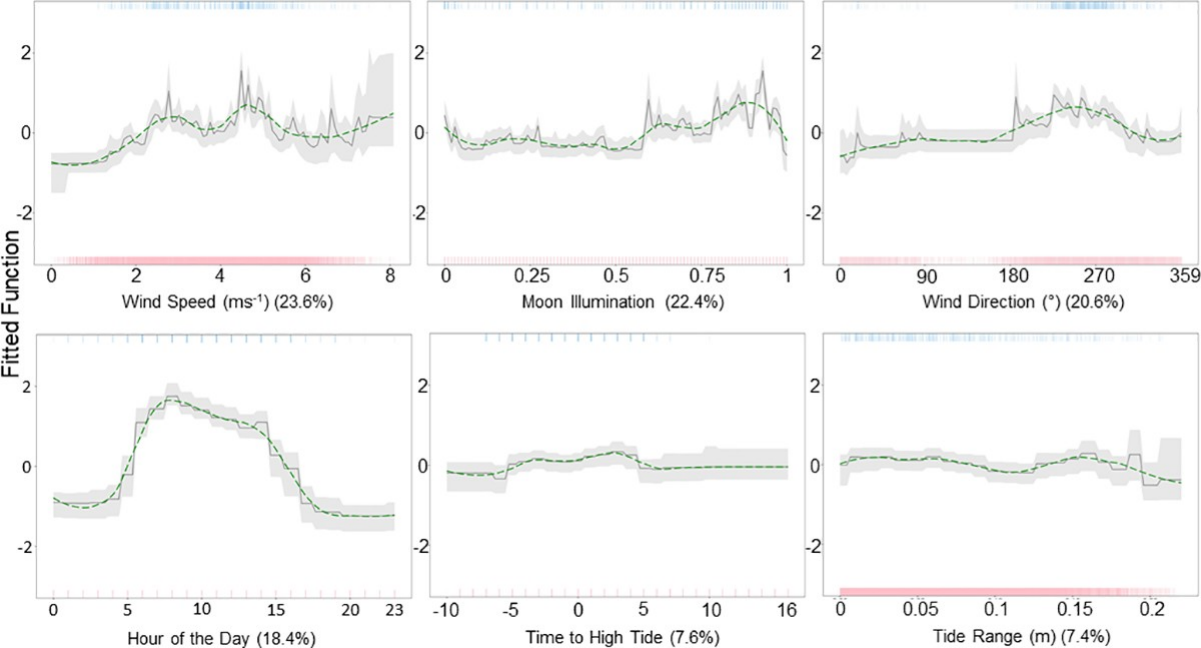
Partial dependency plots for Nelivaru Thila. The effect of each predictor variable on the occurrence of tagged *M*. *alfredi* at Nelivaru Thila, while keeping all other variables at their mean. The green line shows locally weighted smoothing (LOESS). Rugs display the distribution of the data for presence (top, blue), and absence (red, bottom).

The relative influence of the predictor variables (Fig 7) indicates that wind speed (23.6%), moon illumination (22.4%), wind direction (20.6%), and hour of the day (18.4%) are the most accurate predictors of tag detections at Nelivaru Thila. The probability of tag detections increased with moderate wind speed (approximately 4.5 ms^-1^), close to a full moon during the day (with a peak at 8 am). Detection probability also increased when the wind direction was between approximately 255–256° (SSW–W). Less influential variables included time to high tide (7.6%), with the highest detection probability around 3 hours after high tide, with probability generally decreasing with tide range (7.1%).

Significant interactions occurred between wind speed and time to high tide and wind speed and tide range (S3 Table and Fig 5). They suggest that the probability of tag detections increased with high wind speeds (7 – 8ms^-1^), on long flood tides (-8 to -10 hours), and at high wind speeds (7 – 8ms^-1^) when tidal range is greater (approximately 0.15–0.18 m).

### Photographic identification

There was a total of 28373 sightings (<1% of which occurred during the NE Monsoon) of 1835 individually identified *M*. *alfredi* at Hanifaru Bay, Nelivaru Thila, and Dhigu Thila (key habitats) between 2005–2019. Of these, 832 (45%) were males (adult = 703, subadult = 64, juvenile = 65) and 1003 (55%) were female. Twenty-three females were sighted as both a juvenile and an adult; therefore, when summarised by life stage: adult = 496, juvenile = 530. Of the 1835 individuals, 1456 (79%) were sighted more than once, with 1275 (69%) sighted in multiple years and 146 (8%) in 10 or more different years. Of those sighted more than once, 1012 (70%) were sighted at two or more of the key habitats. At Hanifaru Bay, 1805 (98%) of the 1835 individuals were sighted at least once, and 1407 (77%) were sighted on multiple occasions. By demographic group, more male adults have been identified at Hanifaru Bay, while female adults were sighted most frequently (42.8% of sightings) (Table 3).

**Table 3:** Summary of all photo identification records from key habitats by demographics.

	Hanifaru Bay		Dhigu Thila		Nelivaru Thila	
Manta sex and maturity	No. sightings	No. individuals	No. sightings	No. individuals	No. sightings	No. individuals
Female adult	11805	476	350	223	65	56
Female juvenile	7548	518	86	70	18	18
Male adult	7830	685	232	168	51	42
Male juvenile	140	63	4	3	0	0
Male subadult	252	63	4	4	2	2

Total sightings of *M*. *alfredi* and number of individuals identified at the key habitats by demographic group.

At Nelivaru Thila, 118 (6%) of the 1835 individuals were sighted of which, 13 (11%) were sighted on multiple occasions. By demographic group, more female adults have been identified and are most frequently resighted (47.8% of sightings) at Nelivaru Thila (Table 3). At Dhigu Thila, 468 (26%) of the 1835 individuals were sighted at least once, of which 130 (28%) were sighted on multiple occasions. By demographic group, more female adults have been identified and are most frequently resighted (51.8% of sightings) at Dhigu Thila (Table 3).

The mean number of sightings per year was 1892 ± 1239 (range 11–3820), with the highest number recorded in 2018 (3820) (Table 4). In 2019, there were 2904 sightings of 484 individually identified *M*. *alfredi*. Of these, 440 (91%) had been sighted in previous years, and 192 (40%) of these were first sighted at these key habitats a decade or more ago.

**Table 4:** Summary of all photo identification records from the key habitats.

Year	2005	2006	2007	2008	2009	2010	2011	2012	2013	2014	2015	2016	2017	2018	2019	Total No. sightings
No. sightings	11	73	461	2660	2297	3539	871	797	1875	1520	3085	2653	1807	3820	2904	28373
No. Individuals	11	66	317	785	555	708	366	443	542	490	600	589	447	603	484	-

Annual *M*. *alfredi* sightings and the number of individuals identified each year at Hanifaru Bay, Nelivaru Thila, and Dhigu Thila (key habitats).

There was a total of 652 sightings of the 13 tagged *M*. *alfredi* between 2005 and 2019. Of these sightings, 543 (83%) occurred at the three key habitats between 2006–2019, and 541 (99%) of these were during the SW Monsoon. Except for one individual (MV-MA-0414), all of the individuals were sighted on multiple occasions after being tagged, and except for manta MV-MA-0783 (only sighted in Raa Atoll five and eight years after being tagged), all *M*. *alfredi* were sighted at the key habitats in subsequent years after being tagged. For nine of the thirteen *M*. *alfredi*, sightings after they were tagged occurred in multiple years (range = 3–10), with sightings recorded up to 12 years after the individual was tagged (Table 5).

**Table 5:** Summary of tagged manta photo identification records from the key habitats.

	Total no. of sightings per year															
Manta ID	2005	2006	2007	2008	2009	2010	2011	2012	2013	2014	2015	2016	2017	2018	2019	Total
MV-MA-0057				9	6	19		2	5							41
MV-MA-0283		1		8	9	13	3									34
MV-MA-0414			1	4	3											8
MV-MA-0447			2	11	10	9		3	11							46
MV-MA-0783			2													2
MV-MA-0793			2	6	2			1	4		2	5	6	21	10	59
MV-MA-0842			1	2	1	2			1			2				9
MV-MA-0850			1	7	2	4	5		6	5	7	4	4	5	2	52
MV-MA-0901			1	5	10	8	3		14	1	9	11				62
MV-MA-1217				6	25	8		1	15	14	20	6				95
MV-MA-1260				6	7	11	1	1	4	4	7	6	4	5	3	59
MV-MA-1365				5	12	18	1	2	14	3	10					65
MV-MA-1739				1	3	6	1									11
**Total**	0	1	10	70	90	98	14	10	74	27	55	34	14	31	15	543

Annual sightings of tagged *M*. *alfredi* at Hanifaru Bay, Nelivaru Thila, and Dhigu Thila (key habitats). Year tag was deployed is shown with underlining of the total number of sightings in that year.

## Discussion

A combination of photo-ID and acoustic detection data were used to investigate both long-term *M*. *alfredi* visitation trends and small-scale movement patterns to key habitat, predominantly focusing on three aggregation sites (Hanifaru Bay, Nelivaru Thila and Dhigu Thila) on the east side of Baa Atoll.

### Visitation, residency and site fidelity

Photo-ID records suggest variations in visitation patterns may be associated with differences in habitat use by sex and maturity status, which is known to occur for this species in the Maldives and elsewhere [15, 20]. Here, female adults were sighted most frequently at all three key habitats, while a higher number of male adults were identified at Hanifaru Bay. More intensive use of these locations by females than males may be associated with their contrasting mate-seeking behaviour, whereby sexually mature females frequent popular aggregation sites where there is plentiful food, cleaning opportunities and safety, while males move between aggregation sites to find females [12, 15, 58].

Thirteen tagged *M*. *alfredi* were tracked across five locations for between 2–447 days. Diel visitation patterns were observed in the detection data, whereby *M*. *alfredi* were predominantly detected during the day, which is typical of the species [6, 13, 17, 20]. These patterns may be associated with foraging opportunities created by the diel vertical migrations of reef-associated zooplankton, which can become highly enriched in surface waters during daylight hours [59, 60] or because *M*. *alfredi* forage offshore at night [6, 13, 17] Frequenting shallow areas during the day may also be associated with the species’ use of cleaning stations as cleaner fish are only active during daylight hours [34].However, the diel visitation pattern was less pronounced at Dhigu Thila, where more than a third of resident events occurred at night which is likely linked to individuals feeding, or engaging in social interactions or reproductive activity at the location [16, 19].

Residency to the whole acoustic array, measured by the residency index (I_R_), was 29.3%; which was less than 0.5% higher than that measured for only Hanifaru Bay, Nelivaru Thila, Dhigu Thila (henceforth, the key habitat array, I_R_ = 28.9%). Various levels of residency calculated using a similar equation and denoted RI (RI = Number of days detected/Number of days between first and last detection x 100) have been reported in other regions. These range from 65% in the Red Sea [61] to 15% at Lady Elliot Island (LEI) on the Great Barrier Reef [6]. Variations in residency have been attributed to factors such as acoustic array design [20] whereby higher levels of residency may occur when there is more extensive spatial coverage of the study area (e.g. mean RI = 62% [20]). Variations may also be related to the seasonality of the locations; studies reporting relatively high RI appear to be at locations where *M*. *alfredi* exhibit limited or no seasonality [20, 61]. For example, in Seychelles *M*. *alfredi* frequent D’Arros Island and St. Joseph Atoll year-round, although there are seasonal peaks (during the NW Monsoon) in detections [20]. These seasonal peaks suggest that tagged individuals may spend longer at the location during the NW Monsoon, potentially due to enhance food availability induced by monsoon winds [20]. In contrast, detection and photo-ID data here show that key habitat array is only frequented seasonally (during the SW monsoon). These results support previous observations in the Maldives, which suggest that *M*. *alfredi* only utilise locations on the east side of the atolls during the SW Monsoon when winds and concomitant ocean surface currents enhance food availability [7, 30]. Seasonal increases in detections also occurred at LEI where *M*. *alfredi* were suggested to aggregate when sea surface temperature may limit thermal and/or productive habitat availability along the species coastal migration path [6]. Seasonal variations in site use also occurred in West Papua, Indonesia where *M*. *alfredi* RI was also low (21%) [5]. In the current study, the relatively low residency index to the key habitat array is likely related to both the small receiver array size and the seasonality of the location. However, it may also be associated with the sample size of this study [62] or the life stage of the tagged individuals [20]. Here, I_R_ was only considered for 11 of the 13 tagged *M*. *alfredi*, all of which were adult females. Thus, the results do not provide an accurate representation of visitation patterns across all demographics. Future acoustic telemetry studies would benefit from incorporating a larger sample size, which is representative of the population’s demographics, and establishing an acoustic array with greater spatial coverage.

Resident events occurred at each of the five monitored locations. The high utilisation and regular and repeated resident events at key habitat array indicate that the tagged *M*. *alfredi* have a level of fidelity to these three sites. Photo-ID records provide more long-term evidence of site fidelity with some tagged individuals being sighted at the key habitats in multiple subsequent years after their tag had been shed. Site fidelity is a well-reported characteristic of *M*. *alfredi* [7, 10–13], but it can vary considerably between individuals, which is evident from photo-ID data that shows three of the tagged individuals were sighted in 10 or more years between 2007–2019. Records of all sightings also indicate various levels of site fidelity with 1275 (69%) of identified individuals being sighted at the key habitats in multiple years, 11% of which were sighted in ten or more different years. Site fidelity also varied between each site within the key habitat as well as by individual. For example, MV-MA-1271 showed high levels of site fidelity to Hanifaru Bay, but no affinity to Nelivaru Thila. In contrast, MV-MA-1739 and MV-MA-0850 showed much lower levels of site fidelity to Hanifaru Bay, but high levels of site fidelity to Nelivaru Thila and Dhigu Thila, respectively. All three individuals were adult females. Therefore, these variations in site visitation are unlikely to be attributed to differences in habitat use by sex or maturity status. Instead, it is possible that each individual seasonally inhabits a preferred home range, exploiting foraging and cleaning areas within this range, migrating to a secondary home range during the other monsoon. This hypothesis is supported by photo-ID records which show the three *M*. *alfredi* with the lowest number of detections (MV-MA-0414, MV-MA-0783 and MV-MA-0842) were also sighted least frequently at the key habitats, which may indicate they have a different preferred home range to the individuals which were detected (and sighted) more frequently. To further investigate this hypothesis, a much larger receiver array would be required to encompass as many of the known *M*. *alfredi* aggregation sites within the study area, and a much larger, and demographically more representative number of individuals need to be tagged. Employing alternative methods such as satellite telemetry would also be beneficial.

Overall, the high number and extended lengths of resident events of tagged *M*. *alfredi* at Hanifaru Bay indicate that this is the most intensively utilised location, which supports previous studies which have highlighted Hanifaru Bay as a key habitat [7, 15]. In contrast, resident events at both Fares Kandu and Fushifaru Kandu were infrequent and typically short, which indicates that most were recorded when *M*. *alfredi* were cruising within the range of the acoustic receivers rather than utilising the location. At Fares Kandu, almost all resident events occurred during the NE Monsoon or during the transition months between the monsoons. As the location is on the west side of Baa Atoll, these results are consistent with the seasonal pattern in habitat use in the Maldives [7, 15]. One resident event occurred at Fushifaru Kandu, which is located on the east of Lhaviyani Atoll, approximately 60 km from Baa Atoll. The event occurred during the SW Monsoon, which is again consistent with the seasonal pattern in habitat use [7, 15]. Although only a small percentage of the total detections (<0.2%) were recorded at either of these two sites during this study, both are known to be key aggregation sites for *M*. *alfredi* [7]. However, as all individuals during this study were tagged in eastern Baa Atoll, where site fidelity is likely to be high for these individuals, it is probable that the hypothesised secondary (NE Monsoon) home range of each of the tagged individuals are not located at either of these two secondary sites. Indeed, based on subsequent photo-ID data, the identity of many of these sites throughout the archipelago have now been determined [7, 15]. Therefore, any future study should also ensure individuals are tagged at aggregation sites throughout the receiver array. Moreover, only 11 adult female *M*. *alfredi* were tracked for >30 days in this study; thus, a larger sample size, which is more demographically representative, is required to assess visitation patterns more accurately.

### Tag detectability

Two models, one with and without the hourly detection count of the sentinel tag, were compared to investigate the influence environmental variables have on tag detectability [6]. Except for wind speed, the difference in percentage contribution of all variables between models were very low, which suggest that the environmental variables considered here have a very limited influence on the acoustic detectability of tags. For wind speed, the difference in percentage contribution between the models was minor, while a reduction in the strength of the interaction effect that included wind speed was high compared to the interaction strength that did not include wind speed. These results suggest that the contribution of wind speed to the model may have been influenced by a decline in the acoustic detectability of tags with increased wind speeds. Reduced detection probability due to the generation of noise and air bubbles with higher wind speeds that drown out and scatter acoustic signals has been suggested to be a prevalent issue in acoustic studies [63, 64]. There is further evidence of reduced tag detectability in the model that included the hourly detection count of the sentinel tag, which indicated that tag detection probability was lowest when there were <10 detections an hour of the sentinel tag. However, this only occurred in 38 of the 1044 hourly bins. Therefore, it is unlikely to have influenced the BRTs ability to effectively identify the overall predominant influences of *M*. *alfredi* visitation patterns. The BRTs ability to resolve the model despite potential interference with acoustic detectability of tags is also evident from there being no significant interactions between hourly detection count of the sentinel tag and any other variable. These results are based on a subset of data and could become more or less pronounced over a longer period. Here, tag detection probability increased with wind speed in the full Hanifaru Bay model; therefore, the influence of this variable on acoustic reliability appears to be minimal. Nevertheless, without further supporting evidence, the strength of the influence of wind speed has been interpreted with caution, in particular, when tag detection probability declined with increased wind speeds.

### Environmental influences

The drivers of visitation patterns at Hanifaru Bay feeding area, Nelivaru Thila cleaning station, and Dhigu Thila multifunction site (used for both cleaning and feeding) were investigated. Detection probability was highest at Hanifaru Bay with low moon illumination (20–40% of the moon illuminated) and approaching a full moon, just after high tide and into ebb. The influence of the moon at this location is likely to be linked to the lunar effect on tidal intensity [13, 17]. Foraging opportunities at Hanifaru Bay appear to occur when strong lunar tides overcome the force of the prevailing monsoonal current, drawing plankton-rich water from depths outside the atoll back into shallow atoll channels (Hosegood P., unpublished data). Then, in the atoll pass adjacent to Hanifaru Bay (Dharavandhoo Kandu), these currents form a back eddy, trapping and concentrating plankton in the shallow reef inlet (Hosegood P., unpublished data). The increased tidal intensity close to a new and full moon may help to facilitate this process.

Dhigu Thila indicates a different trend to Hanifaru Bay, whereby tag detection probability peaked approaching low tide and increased with tidal range. These tidal conditions may result in less favourable foraging opportunities in Hanifaru Bay, at which time *M*. *alfredi* may utilise cleaning stations [33] at Dhigu Thila. However, Dhigu Thila is a multifunctional site, and the effects of the tide are consistent with those at other locations, where forging *M*. *alfredi* were observed prior to low tide [14, 17] and with a larger tidal range [17]. Therefore, tidal dynamics at Dhigu Thila may be influencing food availability differently to Hanifaru Bay. For example, tidal currents interacting with the steeply rising reef may work to accumulate zooplankton on the upstream side where flow separation can create a pocket of relatively motionless water [65]. Zooplankton retention behind topographic features has previously been observed to occur tidally at *M*. *alfredi* foraging sites [14]. The interaction effect between south-westerly winds and high wind speeds, and time to high tide and wind speed, may represent processes which accelerate these currents. Acceleration of the diverging currents can result in the formation of plankton-rich eddies around abruptly changing topography [65], providing feeding opportunities for *M*. *alfredi*. At Nelivaru Thila, tag detection probability decreased with an increase in tidal range, while it increased during ebb, which is consistent with the observed tidal effects on *M*. *alfredi* cleaning activities in the Coral Sea and at LEI in Australia [17, 66].

Detection probability also increased at Hanifaru Bay with wind speeds of >5 ms^-1^ and with the interaction effect between wind speed >5 ms^-1^ and wind direction of 100–120° (ESE) or 285–350° (W–NNW). These processes may be consistent with the presence of Langmuir Circulation (LC), which may develop throughout the length of Hanifaru Bay at optimal wind speeds of 5 ms^-1^ [67, 68]. Langmuir Circulation describes horizontal counter-rotating cells in the water column, which are induced by the wind and rotate perpendicular to the wind direction [69]. The counter-rotation of alternating cells creates zones of convergence and divergence [70]. Within the convergence zones, zooplankton and other organisms become trapped in highly concentrated bands [71, 72]. These bands and ’slicks’ where the currents converge can be visible on the surface [72]. The interaction effect between high moon illumination and wind speeds >5 ms^-1^ indicate that a combination of high tidal intensity and LC may work together to concentrate zooplankton in Hanifaru Bay, providing ideal foraging conditions for *M*. *alfredi*. Evidence of an association between *M*. *alfredi* visitation patterns at a feeding area and the presence of LC has been reported in the Chagos Archipelago [73]. Evidence of an association between *M*. *alfredi* and LC may also be apparent in other studies. For example, sightings of the species in surface waters at Praia do Tofo in southern Mozambique increased when concentrated bands of zooplankton were visible on the surface [19]. Similarly, *M*. *alfredi* in Komodo Marine Park were observed foraging around surface slicks when there was a high density of particles in the water column [13]. At LEI, studies have found sightings of foraging *M*. *alfredi* and acoustic tag detections increased with wind speeds of approximately 18 km.h^-1^ (5ms^-1^) [6, 17], an optimal speed for the development of LC [67, 68].

In contrast to Hanifaru Bay, detection probability at Nelivaru Thila cleaning station only increased approaching a full moon and at wind speeds where LC is less likely to develop (<5 ms^-1^). It has been suggested that *M*. *alfredi* only engage in cleaning activities when foraging opportunities are limited [33], which could be the case when *M*. *alfredi* visit Nelivaru Thila. Potentially, higher tag detections probability with lower wind speed at Nelivaru Thila was associated with the influence of wind speed on tag detectability. However, other conditions that increased tag detection probability at Nelivaru Thila, such as the interaction effect between the early stages of a flood tide and high wind speed, and high tidal range and high wind speed, suggest that high wind speeds did not cause a great level of interference with tag detections. These conditions also decreased probability at Hanifaru Bay providing further evidence that *M*. *alfredi* visits Nelivaru Thila when foraging opportunities at other sites are limited.

### Conservation concerns and recommendations

*Mobula alfredi* frequently utilised the three key habitats investigated here, which makes the species particularly vulnerable to anthropogenic disturbance at these locations [7, 27, 32]. The effect of disturbance at key habitats in the Maldives has previously been highlighted as a conservation concern [7, 27]. Active protection and enforcement of regulations aimed to minimise disturbance at key aggregation sites currently only exists within Hanifaru MPA [7], which encompasses Hanifaru Bay. However, both Dhigu Thila and Nelivaru Thila remain unmanaged, and Nelivaru Thila unprotected. Furthermore, there are hundreds of other *M*. *alfredi* aggregation sites throughout the Maldives Archipelago, dozens of which also face high and increasing pressures from tourism activities, yet none are currently managed to mitigate these threats [7, 32].

The current study is subject to some limitations. In particular, the small sample size, which was not representative of the overall population demographics, does not allow the investigation of difference in visitation and habitat use by demographics. Moreover, variations in tag detectability could not be assessed at each location; thus, the absence of detection of the tagged individuals does not preclude the presence of the species. However, by combining detection and photo-ID data, the current study provides a limited but valuable insight into habitat use at three key habitats on the east of Baa Atoll. These data allowed some of the small-scale drivers of habitat use at three key habitats to be identified, proposing *M*. *alfredi* may frequent Nelivaru Thila cleaning station when environmental conditions are not suitable for feeding at nearby foraging sites, such as Hanifaru Bay or Dhigu Thila. This suggests that these three locations may form part of a network of key habitats which provide essential services to *M*. *alfredi* in eastern Baa Atoll. Future conservation efforts should focus on extending the current protection measures at Hanifaru MPA to encompass the entire network of key *M*. *alfredi* aggregation sites. Protective measures should also ensure the safe passage of individuals between these sites (migration corridors), where they are also at risk from threats, such as boat strikes and fisheries bycatch [32].

## Supporting information

S1 Table Variance of inflation factor estimates for all predictor variables used for boosted regression tree analysis. (DOCX)

S2 Table Variance of inflation factor estimates for all predictor variables used for tag detectability model. (DOCX)

S3 Table Pairwise interactions between predictor variables. Higher values indicate a stronger interaction effect; near zero indicates negligible interactions. All interactions were significant (*p*<0.01). Subset model of Hanifaru Bay data without (Subset) and with (Subset (S)) hourly detection count of the sentinel tag.
(DOCX)

S1 Fig Hanifaru Bay predictor variables correlation matrix. Spearman’s rank correlation matrix of all predictor variables used for boosted regression tree analysis at Hanifaru Bay.
(TIF)

S2 Fig Dhigu Thila predictor variables correlation matrix. Spearman’s rank correlation matrix of all predictor variables used for boosted regression tree analysis at Dhigu Thila
(TIF)

S3 Fig Nelivaru Thila predictor variables correlation matrix. Spearman’s rank correlation matrix of all predictor variables used for boosted regression tree analysis at Nelivaru Thila
(TIF)

S4 Fig Tag detectability model predictor variables correlation matrix. Spearman’s rank correlation matrix of all predictor variables used for boosted regression tree analysis of the subset of data at Hanifaru Bay including the sentinel tag.
(TIF)

S5 Fig Partial dependency plots for tag detectability BRT. The effect of each predictor variable (while keeping all other variables at their mean) on the occurrence of tagged *M*. *alfredi* at Hanifaru Bay (A) including the hourly sentinel tag detections and (B) without the hourly sentinel tag detections. Grey shading shows 95% confidence interval. Rugs display the distribution of the data.
(TIF)

S6 Fig Interactions between predictor variables for tag detectability BRT. Pairwise interactions between predictor variables while keeping all other variables at their respective mean showing the probability of *M*. *alfredi* tag detections at Hanifaru Bay (A) including the hourly sentinel tag detections and (B) without the hourly sentinel tag detections. All interactions were significant (*p*<0.01).
(TIF)
