# Supplementary material for: Environmental drivers of reef manta ray (Mobula alfredi) visitation patterns to key aggregation habitats in the Maldives
Source: PLoS One. 2021 Jun 23;16(6):e0252470. doi: 10.1371/journal.pone.0252470 (PMC8221513; doi:10.1371/journal.pone.0252470)
Supplement: S2 Table — (DOCX) [file pone.0252470.s002.docx]

| **Predictor Variable** | **Hanifaru Bay** |
| --- | --- |
| Moon Illumination | 1.21 |
| Wind Speed (ms^-1^) | 1.15 |
| Hour of the Day | 1.08 |
| Wind Direction | 1.37 |
| Tide Range (m) | 1.03 |
| Time to High Tide | 1.07 |
| Sentinel Tag | 1.11 |

**S2 Table.** **Variance of inflation factor estimates for all predictor variables used for tag detectability model.**
