# Supplementary material for: Environmental drivers of reef manta ray (Mobula alfredi) visitation patterns to key aggregation habitats in the Maldives
Source: PLoS One. 2021 Jun 23;16(6):e0252470. doi: 10.1371/journal.pone.0252470 (PMC8221513; doi:10.1371/journal.pone.0252470)
Supplement: S3 Table — Higher values indicate a stronger interaction effect; near zero indicates negligible interactions. All interactions were significant (p<0.01). Subset model of Hanifaru Bay data without (Subset) and with (Subset (S)) hourly detection count of the sentinel tag. (DOCX) [file pone.0252470.s003.docx]

**S3 Table**. **Pairwise interactions between predictor variables.** Higher values indicate a stronger interaction effect; near zero indicates negligible interactions. All interactions were significant (*p*<0.01). Subset model of Hanifaru Bay data without (Subset) and with (Subset (S)) hourly detection count of the sentinel tag.

| **Model** | **Predictor 1** | **Predictor 2** | **Interaction size** |
| --- | --- | --- | --- |
| Hanifaru Bay | Wind speed (ms^-1^) | Moon Illumination | 895.9 |
| Hanifaru Bay | Wind speed (ms^-1^) | Wind Direction | 572.5 |
| Subset | Time to High Tide | Moon Illumination | 100.1 |
| Subset | Hour of the Day | Wind speed (ms^-1^) | 129.58 |
| Subset (S) | Time to High Tide | Moon Illumination | 102.3 |
| Subset (S) | Hour of the Day | Wind speed (ms^-1^) | 96.41 |
| Dhigu Thila | Wind speed (ms^-1^) | Time to High Tide | 150.6 |
| Dhigu Thila | Wind speed (ms^-1^) | Wind Direction | 124.1 |
| Nelivaru Thila | Wind speed (ms^-1^) | Tide Range (m) | 178 |
| Nelivaru Thila | Wind speed (ms^-1^) | Time to High Tide | 509 |
