# Supplementary figures and images for: Environmental drivers of reef manta ray (Mobula alfredi) visitation patterns to key aggregation habitats in the Maldives
Source: PLoS One. 2021 Jun 23;16(6):e0252470. doi: 10.1371/journal.pone.0252470 (PMC8221513; doi:10.1371/journal.pone.0252470)

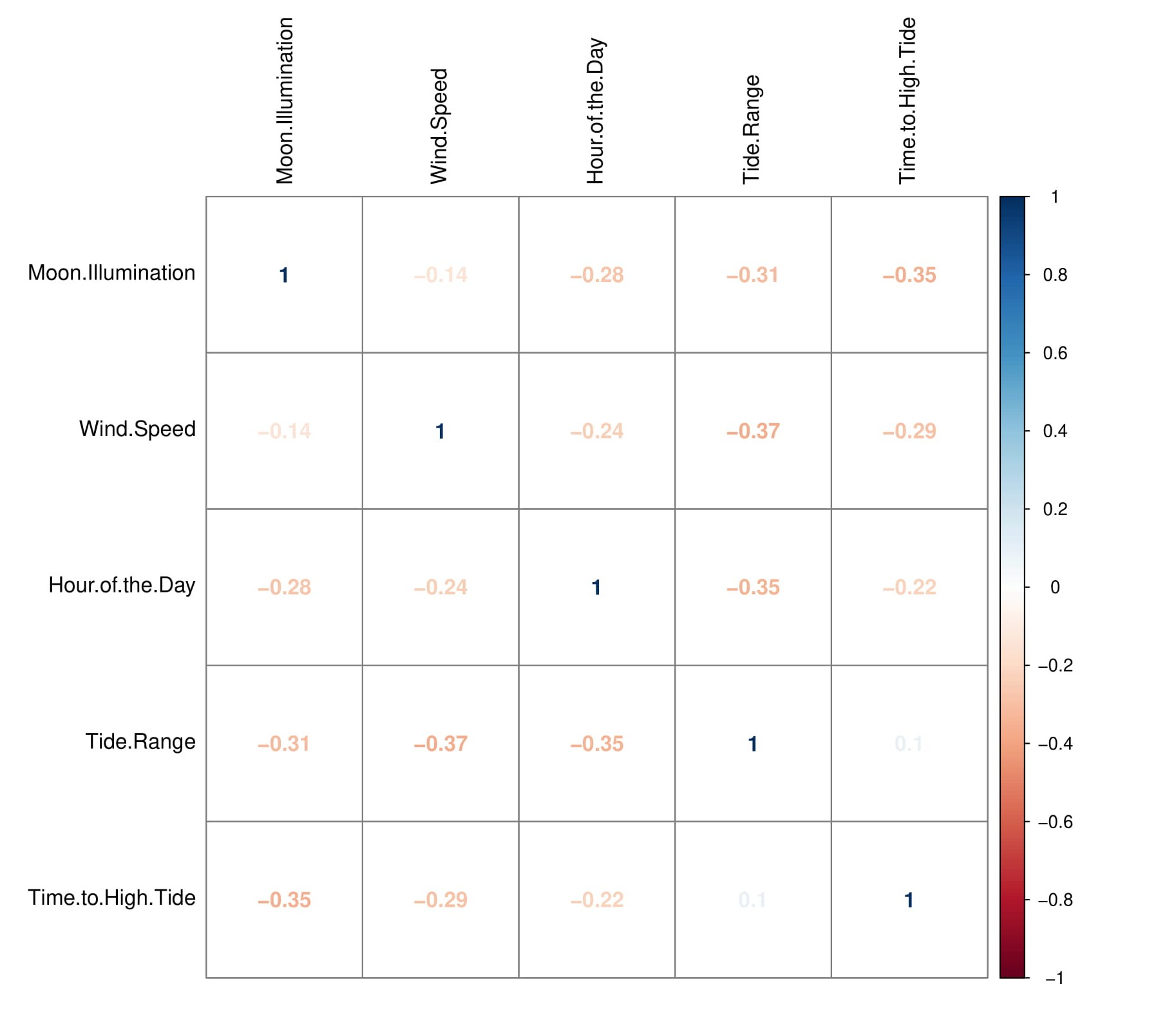

Supplement: S1 Fig — Spearman’s rank correlation matrix of all predictor variables used for boosted regression tree analysis at Hanifaru Bay. (TIF) [file pone.0252470.s004.tif]

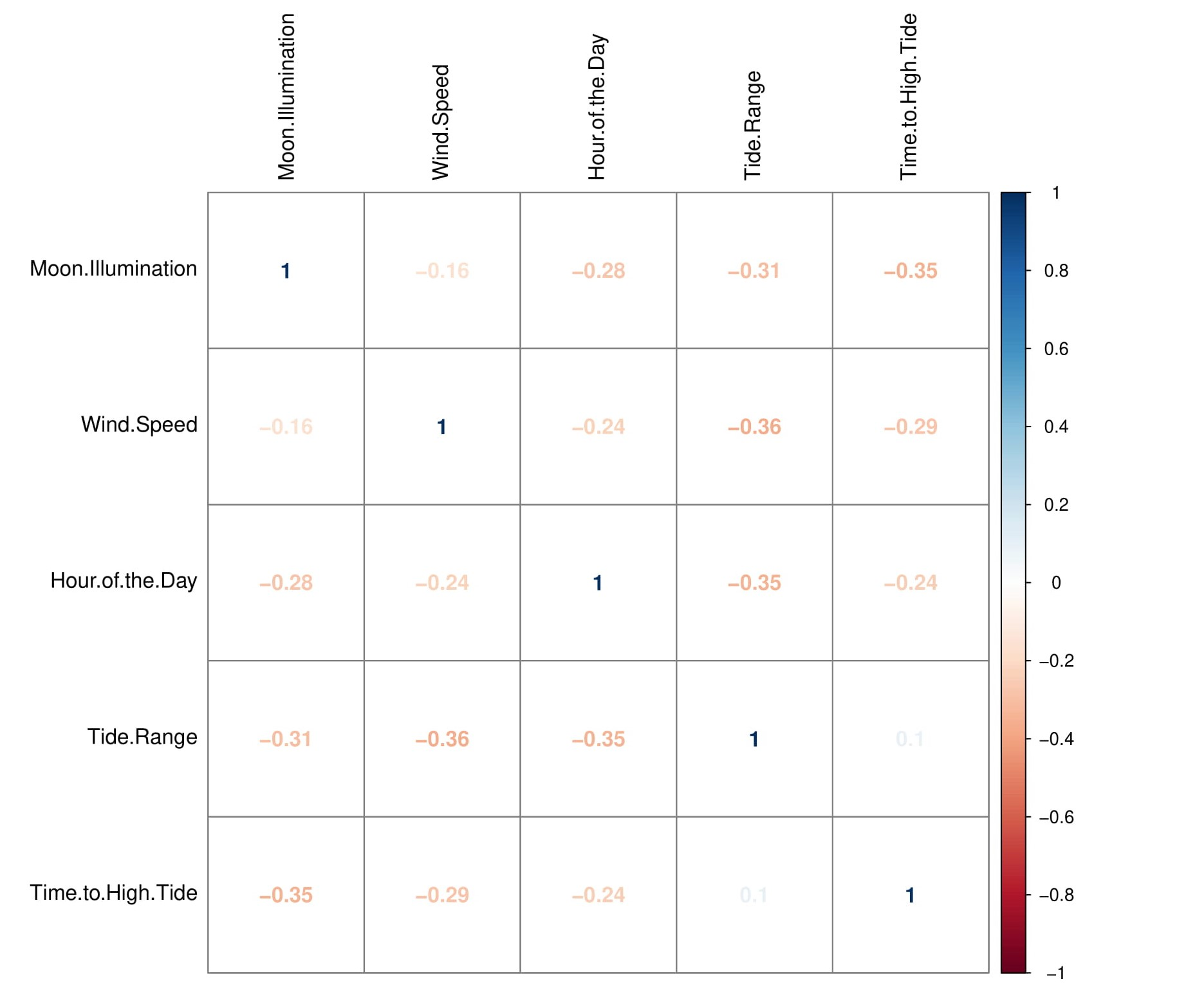

Supplement: S2 Fig — Spearman’s rank correlation matrix of all predictor variables used for boosted regression tree analysis at Dhigu Thila (TIF) [file pone.0252470.s005.tif]

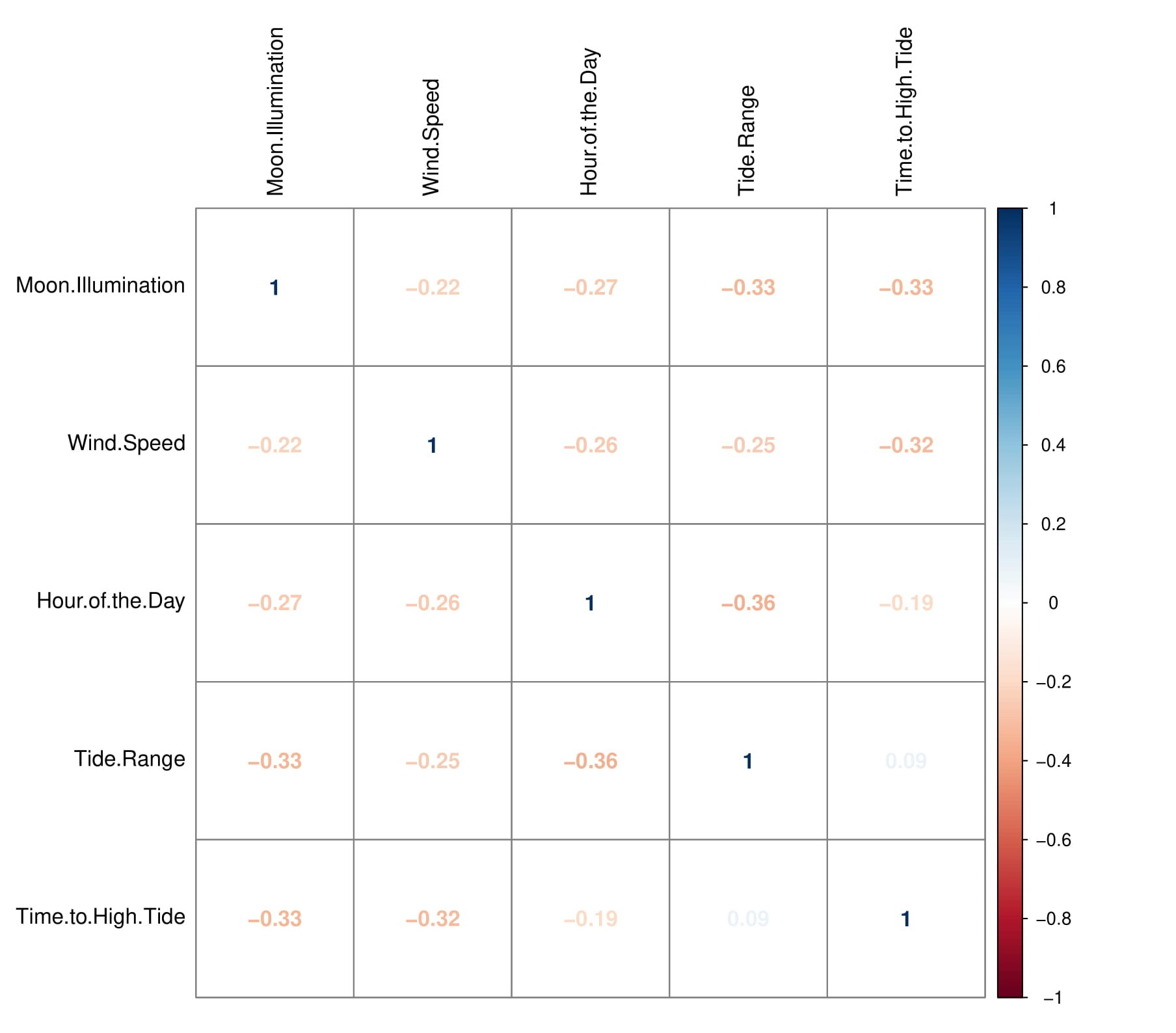

Supplement: S3 Fig — Spearman’s rank correlation matrix of all predictor variables used for boosted regression tree analysis at Nelivaru Thila (TIF) [file pone.0252470.s006.tif]

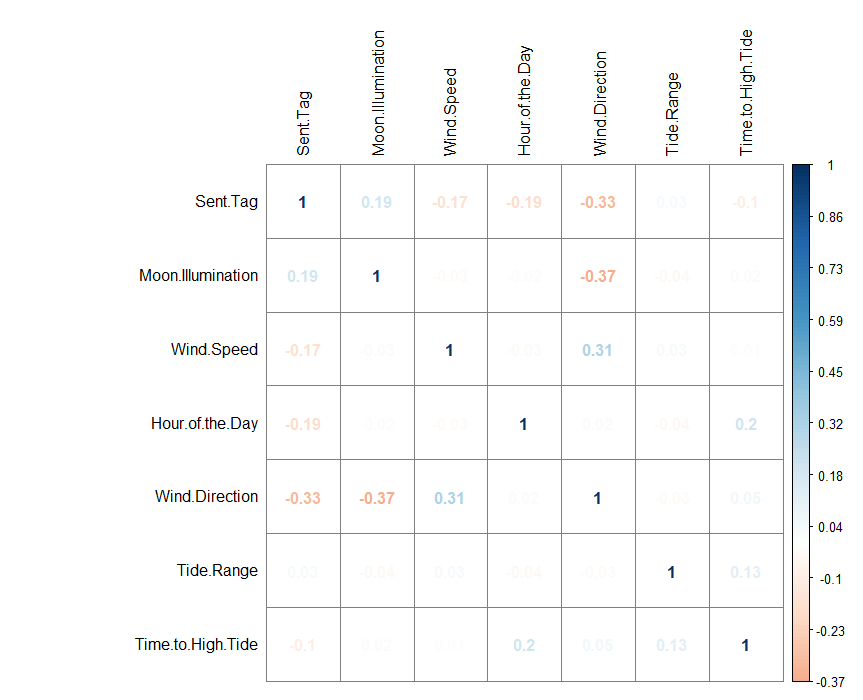

Supplement: S4 Fig — Spearman’s rank correlation matrix of all predictor variables used for boosted regression tree analysis of the subset of data at Hanifaru Bay including the sentinel tag. (TIF) [file pone.0252470.s007.tif]

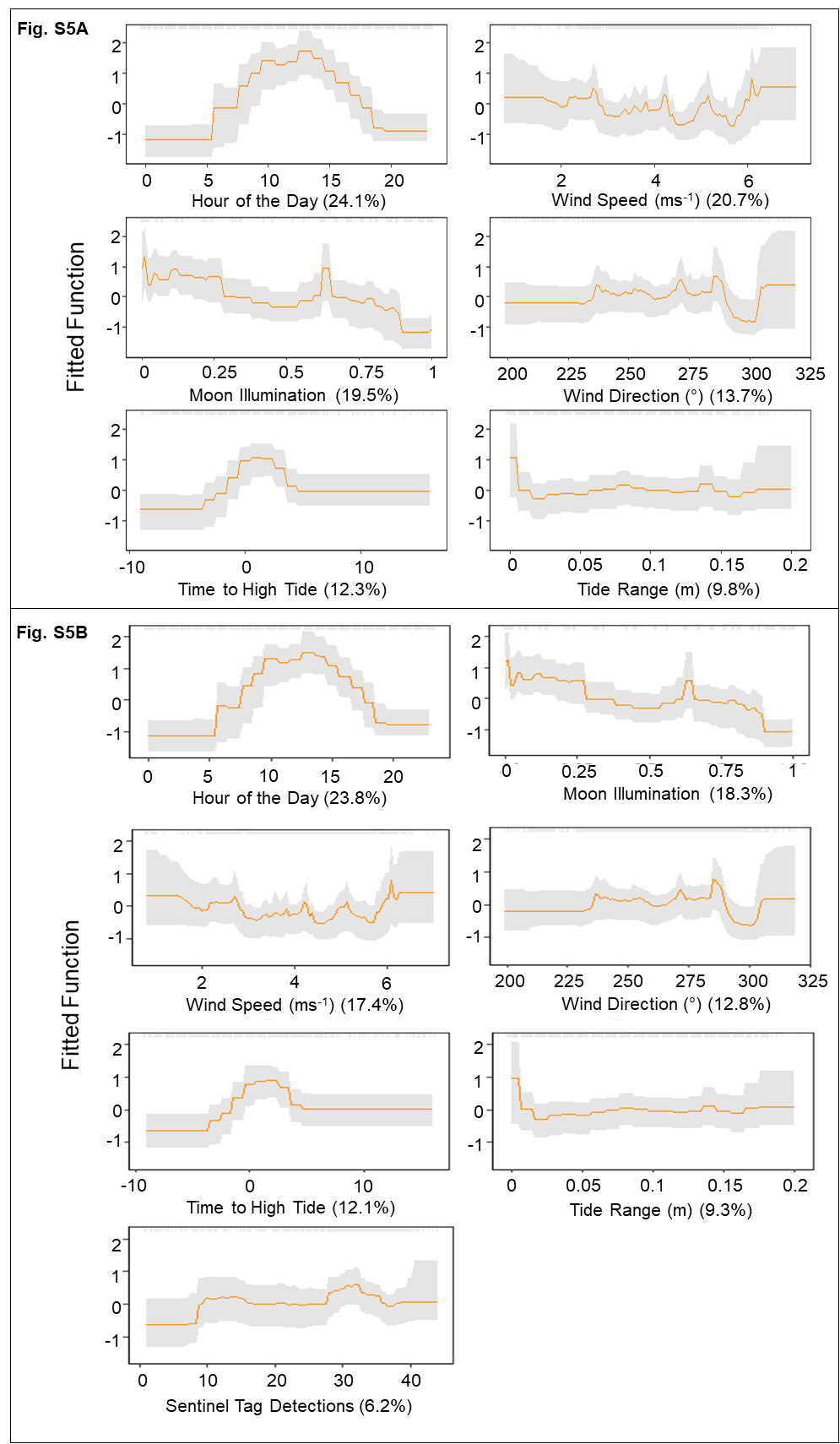

Supplement: S5 Fig — The effect of each predictor variable (while keeping all other variables at their mean) on the occurrence of tagged M. alfredi at Hanifaru Bay (A) including the hourly sentinel tag detections and (B) without the hourly sentinel tag detections. Grey shading shows 95% confidence interval. Rugs display the distribution of the data. (TIF) [file pone.0252470.s008.tif]

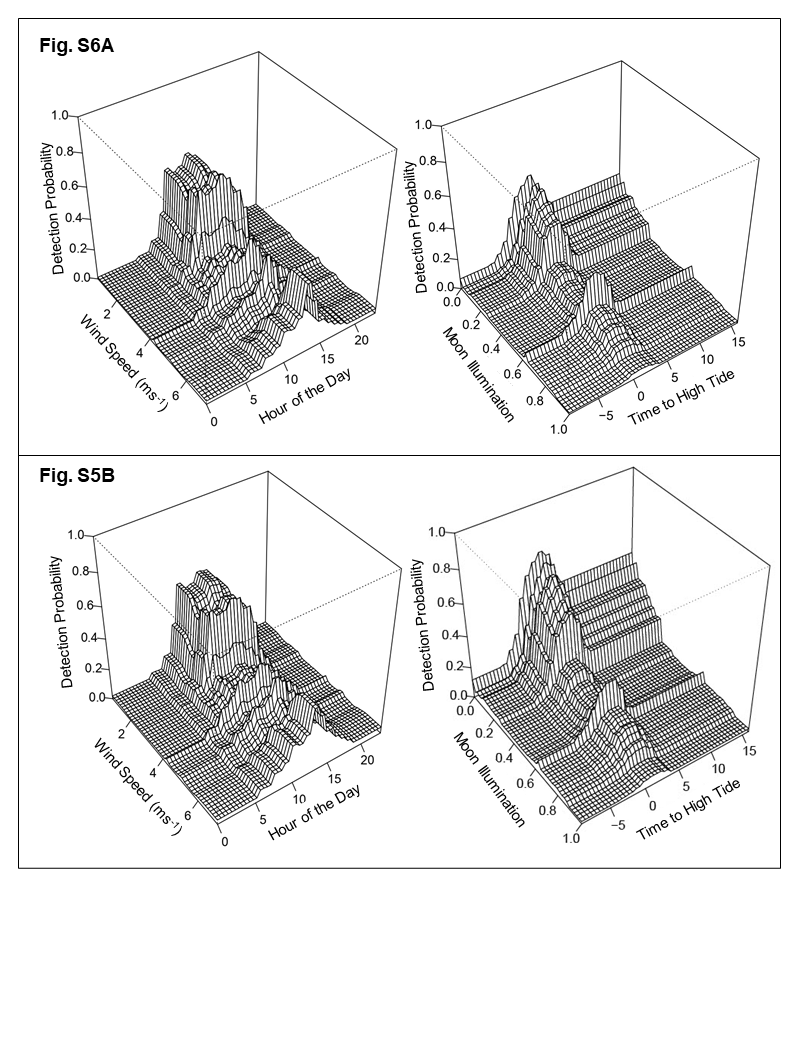

Supplement: S6 Fig — Pairwise interactions between predictor variables while keeping all other variables at their respective mean showing the probability of M. alfredi tag detections at Hanifaru Bay (A) including the hourly sentinel tag detections and (B) without the hourly sentinel tag detections. All interactions were significant (p<0.01). (TIF) [file pone.0252470.s009.tif]
